# Chemical Discrimination of *Astragalus mongholicus* and *Astragalus membranaceus* Based on Metabolomics Using UHPLC-ESI-Q-TOF-MS/MS Approach

**DOI:** 10.3390/molecules24224064

**Published:** 2019-11-09

**Authors:** Yumei Wang, Lei Liu, Yukun Ma, Lina Guo, Yu Sun, Qi Liu, Jicheng Liu

**Affiliations:** The Research Institute of Medicine and Pharmacy, Qiqihar Medical University, Bukui Street 333, Qiqihar 161006, China; yumeiwangqq@163.com (Y.W.); liuleiokc@163.com (L.L.); kuntengchongtian@163.com (Y.M.); gln65@126.com (L.G.); zsy5811321@126.com (Y.S.)

**Keywords:** metabolomics, *Astragalus mongholicus*, *Astragalus membranaceus*, ultra-high performance liquid chromatography coupled with electrospray ionization/quadrupoletime-of-flight mass spectrometry, constituents, discrimination

## Abstract

*Astragalus mongholicus* (MG) and *Astragalus membranaceus* (MJ), both generally known as Huangqi in China, are two perennial herbals widely used in variety diseases. However, there were still some differences in the chemical ingredients between MG and MJ. In this paper, metabolomics combined with the ultra-high performance liquid chromatography coupled with electrospray ionization/quadrupole time-of-flight mass spectrometry (UHPLC-ESI-Q-TOF-MS/MS) was employed to contrastively analyze the chemical constituents between MG and MJ. As a result, principal component analysis showed that MG and MJ were separated clearly. A total of 53 chemical markers were successfully identified for the discrimination of MG and MJ. Of them, the contents of 36 components including Astragaloside I~III, Astragaloside IV, Agroastragaloside I, etc. in MJ were significantly higher than those in MG. On the contrary, the contents of 17 other components including coumaric acid, formononetin, sophoricoside, etc. in MG were obviously higher than those in MJ. The results showed that the distinctive constituents in MG and MJ were remarkable, and MJ may own stronger pharmacological activities than MG. In a word, MG and MJ may be treated as two different herbs. This paper demonstrated that metabolomics was a vitally credible technology to rapidly screen the characteristic chemical composition of traditional Chinese medicine.

## 1. Introduction

Traditional Chinese medicine (TCM), which originated in ancient China, has played a positive role in Chinese people’s health for thousands of years. Besides, with its continuation and development, TCM has been extensively applied for diseases’ prevention and treatment not only in China, but also in some Western countries. *Astragalus mongholicus* (locally known as menggu huangqi, MG) and *Astragalus membranaceus* (locally known as mojia huangqi, MJ), two leguminous plants belonging to the same family of TCM named Huangqi in Chinese Pharmacopoeia, were generally used to improve immunity [1], cardiotonic [2], anti-hypertension [3,4], anti-cancer [4], anti-virus [5], and anti-diabetes [6], among others [7,8,9,10]. MG and MJ share great similarities in morphology, chemical constituent, and gene sequence [11,12]. The taxonomy controversy of MG and MJ has lasted several decades. As the discrimination of TCM played a critical role in pharmacological and clinical effects, it was urgent to find a feasible method for discriminating MG and MJ. At present, MG was considered as a variation of MJ, as the main constituents of MG and MJ were similar, generally including flavonoids, saponins, polysaccharides, and so on [13]. However, there were still many differences in the detailed chemical composition between the two herbs.

Metabolomics, an important “omics” technology in system biology, is a comprehensive method focusing on high-throughput qualitative and quantitative analysis of small molecular (<1000 Da) metabolites including lipids, amino acids, nucleic acids, organic acids, vitamins, peptides, thiols, carbohydrates, et al. Metabolites, the products of metabolic synthesis, were deemed as the effective material basis in metabolic study. At present, metabolomics has been widely applied in modern studies, including disease diagnosis [14], drug toxicity evaluation [15], microbial species classification [16], plant metabolism [12], and food nutrition [17,18], among others [16,17,18,19,20], in various Chinese herbs such as *Astragalus membranaceus* [21], *Huperzia serrata* [22], *Panax quinquefolius* [23], chrysanthemum cultivars [24,25], and so on [25,26,27]. Therefore, metabolomics may become a promising tool for resolving the difference and mechanisms of TCM.

**Figure 1:**
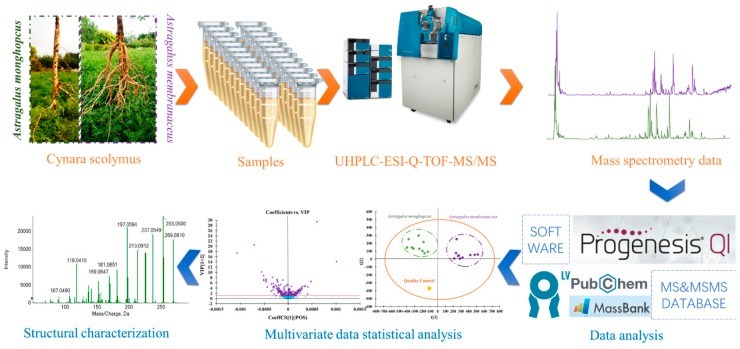
The experimental procedures for chemical discrimination of *Astragalus mongholicus* (MG) and *Astragalus membranaceus* (MJ).

In this paper, in order to solve the taxonomy uncertainty and explore the discrimination of MG and MJ, metabolomics method based on ultra-high performance liquid chromatography coupled with electrospray ionization/quadrupole time-of-flight mass spectrometry (UHPLC-ESI-Q-TOF-MS/MS) technology was employed to dramatically distinguish MG and MJ. The experimental detail procedures are shown in Figure 1, which was also provided as supplementary materials (Figure S1). Our present study indicated that metabolomics was a powerful approach for discriminating different Chinese herbs. The obtained results not only provided chemical information for their quality assessment, but also offered rigorous evidence for further pharmacological study of MG and MJ.

## 2. Results and Discussion

### 2.1. Selection of Extraction Methods

Before the formal analysis of MG and MJ, extraction methods were optimized in order to gain satisfying chromatographic peaks. By testing different extraction solvents (50% methanol, 75% methanol, and 100% methanol) and diverse extraction times (0.5 h, 1 h, 1.5 h, 2 h), the method of extracting for 1 h with 75% methanol showed the large number of detectable peaks.

### 2.2. Optimization of UHPLC-ESI-Q-TOF-MS/MS Conditions

**Figure 2:**
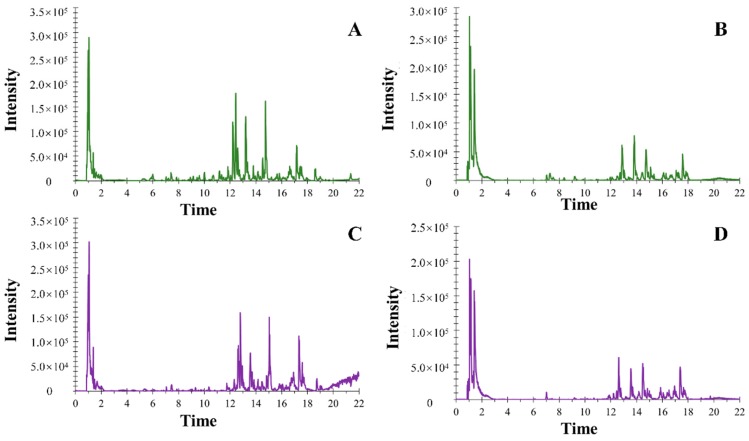
The base peak chromatograms (BPCs) in positive mode (**A**: MG; **B**: MJ) and negative mode (**C**: MG; **D**: MJ).

The employed UHPLC-ESI-Q-TOF-MS/MS was a powerful technology, which could offer integrate information of TCM. Therefore, the conditions of UHPLC-ESI-Q-TOF-MS/MS were very important for the plant metabalomics analysis. In our paper, the mobile phase was optimized and 0.1% formic acid in water (A) and 0.1% formic acid in acetonitrile (B) showed a satisfying result. Besides, two columns including ACQUITY™ BEH C_18_ column (100 mm × 2.1 mm i.d., 1.8 µm) and ACQUITY™ UPLC HSS T_3_ column (100 mm × 2.1 mm i.d., 1.8 μm) were explored during the analysis, and the latter one showed much more approving data. Furthermore, in order to obtain good sensitivity and resolution, the flow rate was set at 0.4 mL/min and the injection volume was selected at 3 µL. Besides, MS conditions, including ion spray voltage; ESI heater temperature; and pressures of GS1, GS2, and CUR were optimized respectively so as to achieve better separation and stronger response for most peaks from MG and MJ. Anything else, in order to accelerate the efficiency of metabalomics analysis, a characteristic acquire mode IDA was employed to gain the MS/MS information of ions when acquiring MS information. On this basis, the top 10 ions during every 0.08 s accumulation time interval were the most meaningful ions matched IDA criteria. Besides, the dynamic background subtraction method was used to differentiate the background and matrix-related MS/MS ions intelligently. On the basis of the above conditions, the majority of base peak chromatograms (BPCs) were separated well (Figure 2), indicating that the established method was suitable for our analysis. 

### 2.3. Characteristic Multivariate Data Analysis of Metabalomics

**Figure 3:**
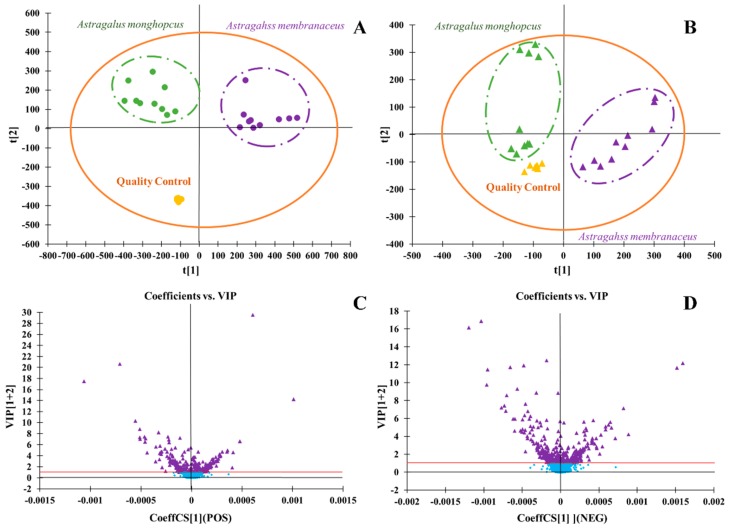
The analysis of MG and MJ samples by UHPLC-ESI-Q-TOF-MS/MS. (**A**)-The principal component analysis (PCA) scores plots in positive ion mode; (**B**)-the PCA scores plots in negative ion mode; (**C**)-the variable importance in the projection (VIP) plot of the ions whose value ≥1 in positive ion mode; (**D**)-the VIP plot of the ions whose value ≥1 in negative ion mode.

PCA and OPLS-DA analysis were used in the multivariate data analysis. PCA is an unsupervised pattern recognition that could effectively find out the most important information in data by reducing the original complex data. It could be used for variable reduction and separation into different classes. In this paper, the PCA model was used to identify the difference of metabolites from the MG group and MJ group. The PCA score maps were shown in Figure 2, which was also be provided as supplementary materials (Figure S2). OPLS-DA was used to discriminate between the MG group and MJ group. As shown in Figure 3, which was also provided as supplementary materials (Figure S3), the MG group and MJ group were clearly divided into two regions, indicating that there were significant chemical differences between the MG group and MJ group, and the established metabolomics method could characterize the chemical characteristics successfully. VIP was a common method for evaluating the contribution of variables in OPLS-DA. It was generally believed that the ions whose value ≥1 owned statistical significance and indicated the characteristics of study object. As shown in the VIP-plot in Figure 3, ions whose VIP value ≥1 were selected and regarded as the most significant differential chemical markers of the MG group and MJ group.

### 2.4. Identification of Chemical Markers between MG and MJ

**Figure 4:**
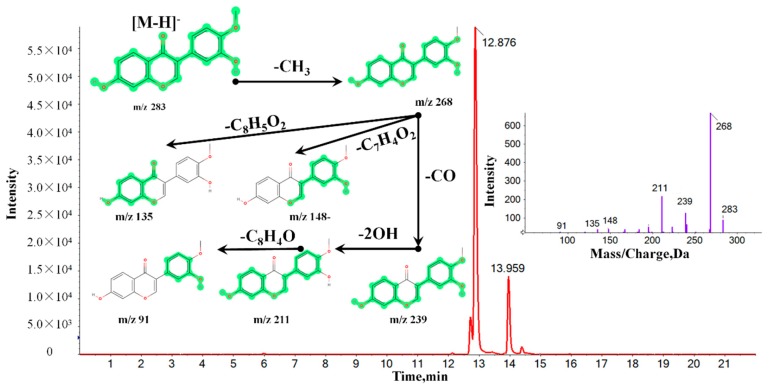
The MS and MS/MS information of Calycosin detected in the MG group by UHPLC-ESI-Q-TOF-MS/MS.

On the basis of the VIP results, the candidate ions between MG and MJ were identified tentatively. Take the identification of Calycosin for example. In positive mode, the ion (RT = 12.88 min and [M − H]^−^ = 283.0688) detected in the MG group was calculated to be C_16_H_12_O_5_ based on the elemental composition and fractional isotope abundance. Besides, the degree of unsaturation was calculated as 14, indicating that it may be a compound with one or two benzene rings. The main MS/MS fragments ions were *m*/*z* 267, *m*/*z* 239, *m*/*z* 211, and *m*/*z* 91, meaning that the fragments may be C_15_H_7_O_5_^−^, C_14_H_7_O_4_^−^, C_13_H_7_O_3_^−^, and C_6_H_3_O^−^, respectively. Finally, after being contrasted with the MS/MS fragments database, the ion was finally confirmed to be Calycosin. The corresponding mass spectrums and related structures were shown in Figure 4, which was also provided as supplementary materials (Figure S4). According to the above-mentioned analysis method, a total of 53 chemical markers discriminating MG and MJ were successfully identified, including 27 candidate ions in positive mode and 26 candidate ions in negative mode. The detailed information of identified components was shown in Table 1, the related MS/MS maps were also provide as supplementary materials (peak 1-53).

### 2.5. Relative Intensity Comparison of Chemical Markers between MG and MJ

**Figure 5:**
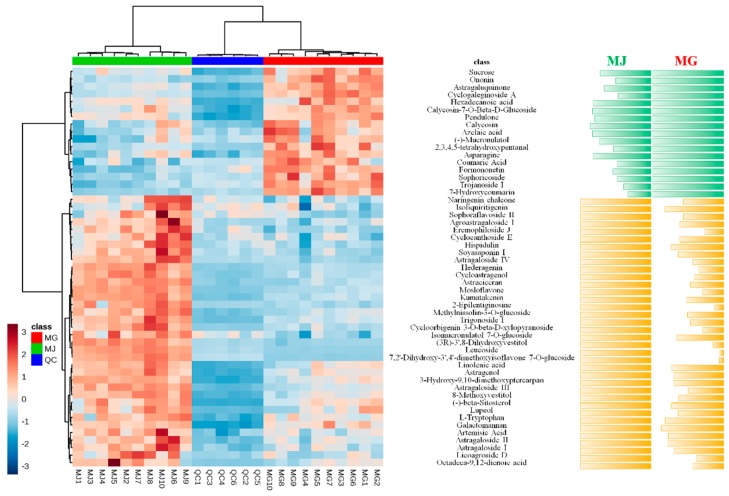
The heat map analysis and detail relative content comparison of chemical markers between MG and MJ.

Metabolomics was a high-throughput method to analyze the different metabolic components in plant extracts. It could clarify the primary and secondary metabolites on different levels and provide a reliable basis for the quality control of Chinese herbs. On the basis of the metabolomics analysis, heat maps were employed to show the relative intensity comparison of chemical markers between MG and MJ. Ulteriorly, a relative content comparison between MG and MJ was also conducted in order to reveal the content of chemical markers intuitively. The detailed results were shown in Figure 5, which was also be provided as supplementary materials (Figure S5).

As shown in Figure 5, MJ showed higher levels of 36 components, including Naringenin chalcone, Isoliquiritigenin, Sophoraflavoside II, Agroastragaloside I, Eremophiloside J, Cyclocanthoside E, Hispidulin, Soyasaponin I, Astragaloside IV, Hederagenin, Cycloastragenol, Astraciceran, Mosloflavone, Kumatakenin, 2-Epilentiginosine, Methylnissolin-3-*O*-glucoside, Trigonoside I, Cycloorbigenin 3-*O*-beta-d-xylopyranoside, Isomucronulatol 7-*O*-glucoside, (3R)-3′,8-Dihydroxyvestitol, Leucoside, 7,2′-Dihydroxy-3′,4′-dimethoxyisoflavone 7-*O*-glucoside, Linolenic acid, Astragenol, 3-Hydroxy-9,10-dimethoxyptercarpan, Astragaloside III, 8-Methoxyvestitol, (+)-beta-Sitosterol, Lupeol, L-Tryptophan, Galactomannan, Artemisic Acid, Astragaloside II, Astragaloside I, Licoagroside D, and Octadeca-9,12-dienoic acid. On the contrary, MG showed higher levels of 17 other components, including Sucrose, Ononin, Astragaluquinone, Cyclogaleginoside A, Hexadecanoic acid, Calycosin-7-*O*-Beta-d-Glucoside, Pendulone, Calycosin, Azelaic acid, (−)-Mucronulatol, 2,3,4,5-tetrahydroxypentanal, Asparagine, Coumaric Acid, Formononetin, Sophoricoside, Trojanoside I, and 7-Hydroxycoumarin.

Traditionally, MG and MJ were used interchangeably in clinical because they were considered to share the equal efficacy. However, MG and MJ were two species of *Astragalus* usually planted in different areas. According to great different growing locations and climate conditions, numerous chemical wispy differences were inevitable between MG and MJ. The identified chemical components showed entirely different activities in MG and MJ. Among the chemical components, 68% of them were higher in MJ than in MG. Besides, four primary saponins constituents including Astragaloside I, Astragaloside II, Astragaloside III, and especially Astragaloside IV with anti-cancer [28], anti-oxidant [29], anti-inflammatory [30], immune regulation [31], and metabolic regulation activities [32], among others [33,34,35], in MJ were distinctly higher than that in MG. The result offered evidence that MJ may own stronger pharmacological activity and wider application than MG. Moreover, even if MG and MJ were applied in the same disease, they may be attributed to different constituents and mechanisms.

## 3. Materials and Methods

### 3.1. Plant Materials, Reagents and Chemicals

The radix of MG (age of 2) and MJ (age of 2) were both collected from the medical plants garden in Qiqihar Medical University and identified by doctor Jicheng Liu from Qiqihar Medical University.

Methanol and acetonitrile (HPLC grade) were bought from Merck company (Darmstadt, Germany). Formic acid (HPLC grade) was bought from Thermo Fisher Scientific (Pittsburgh, PA, USA). Distilled water was purified by a Milli-Q pure-ultrapure water system (Millipore, Bedford, MA, USA). Other reagents and chemicals were of analytical grade. 

### 3.2. Preparation of MG Extraction and MJ Extraction for UHPLC-ESI-Q-TOF-MS/MS Analysis

A total of 1 g of dried MG and MJ powder was respectively steeped in 15-fold volume of 75% methanol in a reflux devices and extracted twice, each extraction lasting for 1 h. Then, the supernatant was obtained and filtered through a 0.22 μm membrane. Finally, a 3 µL aliquot of MG and MJ sample solution was injected for UHPLC-ESI-Q-TOF-MS/MS analysis.

### 3.3. Conditions of Analysis Platform

An ultra-high performance liquid chromatography system LC-30A (Shimadzu Corporation, Kyoto, Japan) coupled with a mass spectrometer Triple TOF 4600 system (AB Sciex Corporation, Redwood city, CA, USA) equipped with an electrospray ionization (ESI) source was employed in the analysis. The parameters of liquid chromatography and mass spectrometry were set as follows.

**Chromatographic conditions:** An ACQUITY™ UPLC HSS T_3_ column (100 mm × 2.1 mm i.d., 1.8 μm, Waters Corporation, Milford, MA, USA) was used for the separation at 35 °C. The optimal mobile phase consisted of 0.1% formic acid in water (A) and 0.1% formic acid in acetonitrile (B). The linear gradient program was set as follows: 0.01–3 min, 1–10% B; 3–9 min, 10–30% B; 9–18 min, 30–100% B; 18–22 min, 100–100% B. The flow rate was maintained at 0.4 mL/min and the injection volume was set at 3 uL. On the basis of the above conditions, the chromatographic peaks of MG and MJ were detected satisfactorily.

**Mass spectrometry conditions:** In positive mode, ionspray voltage floating (ISVF) was set at 5500 V; ESI heater temperature was maintained at 600 °C; nebulizer gas (GS 1), auxiliary gas (GS 2), and curtain gas (CUR) were set at 55 psi, 55 psi, and 30 psi, respectively; declustering potential (DP) and collision energy (CE) were set at 100 V and 10 V. The accumulation time of TOFMS was set at 0.15 s. In negative mode, ISVF was set at −4500 V; other parameters were the same as in positive mode. During the positive and negative acquire mode, MS and MS/MS data were acquired simultaneously via an information-dependent acquisition (IDA) mode. Under the IDA-MS/MS experiment, CE was set at 40 ± 20 V, and at most, 10 candidate ions were monitored per cycle. Besides, a dynamic background subtract mode, which distinctly differentiated the background and matrix-related MS/MS ions from the endogenous or exogenous components, was conducted during the analysis. Finally, a continuous calibration was proceeded every four injections during the analysis period.

### 3.4. Multivariate Data Processing

An integrated software Progenesis QI (Nonlinear Dynamics, version: 2.4, Waters Corporation, Milford, MA, USA) containing EZinfo 3.0 was employed for the discovery and identification of potential chemical markers. Firstly, the obtained original data files (.raw) of MG and MJ were formatted to new data files (wiff. and wiff. Scan) via Progenesis QI software; three loading forms including [M + H]^+^, [M + Na]^+^, and [M + NH_4_]^+^ were selected for the positive ion mode and two loading forms including [M − H]^−^ and [M + FA − H]^−^ were selected for the negative ion mode. After peak alignment, peak matching, and peak extraction, a three-dimensional matrix including retention time, *m*/*z*, and peak area information was obtained successfully. Next, it was exported into EZinfo 3.0 software for further data analysis, after pareto scaling transformation, principal component analysis (PCA), and orthogonal partial least squares discriminant analysis (OPLS-DA) analysis were conducted to further recognize the pattern of the data. Then, variable importance in the projection plots (VIP-plots) were constructed to choose the differential variables. At last, interest variables whose VIP ≥1 were listed to the constructed OPLS-DA and the differential ions between MG and MJ were finally screened out. 

### 3.5. Identification of Potential Chemical Markers

The above-mentioned differential ions were transferred back to Progenesis QI software and marked with tag I, meanwhile, ions whose *p*-value (as measured by Student’s t-test) less than 0.05 were marked with tag II. Then, tag I and tag II were further subjected to matching with some databases such as SDF database created by the information of *Astragalus* plant in the SciFinder database and MS/MS database (Natural product database from AB Sciex, Metlin database, and National Institute of Standards and Technology database). On the basis of the characteristic fragments and the fragmentation patterns confirmed by MS/MS, the list of identified potential chemical markers, including compound’s formulas, molecular weights, compound’s names, Chemical Abstracts Service numbers, and mass errors, were finally obtained.

## 4. Conclusions

In this paper, a powerful approach, UHPLC-ESI-Q-TOF-MS/MS, based on metabolomics combined with a multivariate statistical analysis method was employed to successfully discriminate two Chinese herbs MG and MJ. As a result, MG and MJ could be obviously separated in a PCA loading plot, and a total of 53 chemical markers discriminating MG and MJ were screened out and identified successfully. Of them, the contents of 36 components including Naringenin chalcone, Isoliquiritigenin, Sophoraflavoside II, Agroastragaloside I, Eremophiloside J, Cyclocanthoside E, Hispidulin, Soyasaponin I, Astragaloside IV, Hederagenin, Cycloastragenol, Astraciceran, Mosloflavone, Kumatakenin, 2-Epilentiginosine, Methylnissolin-3-*O*-glucoside, Trigonoside I, Cycloorbigenin 3-*O*-beta-d-xylopyranoside, Isomucronulatol 7-*O*-glucoside, (3R)-3′,8-Dihydroxyvestitol, Leucoside, 7,2′-Dihydroxy-3′,4′-dimethoxyisoflavone 7-*O*-glucoside, Linolenic acid, Astragenol, 3-Hydroxy-9,10-dimethoxyptercarpan, Astragaloside III, 8-Methoxyvestitol, (−)-beta-Sitosterol, Lupeol, L-Tryptophan, Galactomannan, Artemisic Acid, Astragaloside II, Astragaloside I, Licoagroside D, and Octadeca-9,12-dienoic acid in MJ were significantly higher than those in MG. On the contrary, the contents of 17 other components including Sucrose, Ononin, Astragaluquinone, Cyclogaleginoside A, Hexadecanoic acid, Calycosin-7-*O*-Beta-d-Glucoside, Pendulone, Calycosin, Azelaic acid, (−)-Mucronulatol, 2,3,4,5-tetrahydroxypentanal, Asparagine, Coumaric Acid, Formononetin, Sophoricoside, Trojanoside I, and 7-Hydroxycoumarin in MG were obviously higher than those in MJ. The above-mentioned chemical markers discriminated the two herbal medicines successfully. Summing up the above, MJ may own wider application and stronger pharmacological activities than MG. The results not only provided helpful chemical information for quality assessment and mechanism research between MG and MJ, but also offered a meticulous selection for different diseases in clinical.

## Figures and Tables

**Table 1:** The detailed information of identified components in positive ion mode and negative ion mode byUHPLC-ESI-Q-TOF-MS/MS.

No.	Rt	*m*/*z*	CAS&Pubchem ID	Identification	Adducts	Formula	Error (ppm)	*p*-Value
1	1.01	132.0541	3130-87-8	Asparagine	M − H_2_O − H, M − H	C_4_H_8_N_2_O_3_	4.74	3.7 × 10^−4^
2	1.05	195.0513	70849-23-9	2,3,4,5-tetrahydroxypentanal	M + FA − H	C_5_H_10_O_5_	2.15	2.0 × 10^−5^
3	1.10	342.1158	57-50-1	Sucrose	M − H, M + FA − H	C_12_H_22_O_11_	−1.09	1.7 × 10^−4^
4	7.43	203.0829	73-22-3	L-Tryptophan	M − H	C_11_H_12_N_2_O_2_	1.58	6.1 × 10^−3^
5	10.23	161.0249	93-35-6	7-Hydroxycoumarin	M − H	C_9_H_6_O_3_	3.09	3.4 × 10^−10^
6	11.37	209.0460	25429-38-3	Coumaric Acid	M + FA − H	C_9_H_8_O_3_	3.00	7.7 × 10^−5^
7	11.72	431.0987	152-95-4	Sophoricoside	M−H	C_21_H_20_O_10_	0.77	1.5 × 10^−6^
8	12.88	283.0608	20575-57-9	Calycosin	M − H	C_16_H_12_O_5_	−1.52	2.5 × 10^−3^
9	12.91	447.1312	464196-55-2	Licoagroside D	M − H	C_22_H_24_O_10_	3.46	4.2 × 10^−3^
10	13.02	187.0979	66923-62-4	Azelaic acid	M − H	C_9_H_16_O_4_	1.61	2.9 × 10^−3^
11	13.40	299.0560	1447-88-7	Hispidulin	M − H	C_16_H_12_O_6_	−0.47	1.8 × 10^−5^
12	13.76	255.0663	961-29-5	Isoliquiritigenin	M − H	C_15_H_12_O_4_	0.07	2.3 × 10^−2^
13	13.76	463.1618	94367-43-8	Isomucronulatol 7-*O*-glucoside	M − H	C_23_H_28_O_10_	1.71	3.3 × 10^−6^
14	13.82	267.0658	485-72-3	Formononetin	M − H	C_16_H_12_O_4_	−1.66	9.1 × 10^−09^
15	14.09	971.4902	147540-80-5	Sophoraflavoside II	M − H	C_48_H_76_O_20_	4.61	1.2 × 10^−3^
16	14.42	271.0614	25515-46-2	Naringenin chalcone	M − H	C_15_H_12_O_5_	0.63	9.0 × 10^−4^
17	14.42	831.4781	170969-74-1	Cyclocanthoside E	M + FA − H	C_41_H_70_O_14_	4.21	4.1 × 10^−4^
18	14.85	827.4408	1037218-20-4	Eremophiloside J	M + FA − H	C_41_H_66_O_14_	−1.67	1.5 × 10^−6^
19	14.89	784.4650	84687-43-4	Astragaloside IV	M − H, M + FA − H	C_41_H_68_O_14_	4.28	2.6 × 10^−8^
20	15.17	942.5210	51330-27-9	Soyasaponin I	M − H, M + FA − H	C_48_H_78_O_18_	2.30	1.3 × 10^−5^
21	15.25	871.4705	84676-89-1	Astragaloside II	M + FA − H	C_43_H_70_O_15_	0.96	6.0 × 10^−4^
22	15.35	301.1075	20878-97-1	(−)-Mucronulatol	M − H	C_17_H_18_O_5_	−2.23	4.2 × 10^−4^
23	15.51	915.4903	156769-94-7	Agroastragaloside I	M + FA − H	C_45_H_74_O_16_	−4.90	1.9 × 10^−4^
24	15.60	909.4863	386273-42-3	Trojanoside I	M − H	C_47_H_74_O_17_	1.04	3.0 × 10^−9^
25	16.10	868.4847	84680-75-1	Astragaloside I	M + Cl, M + FA − H	C_45_H_72_O_16_	3.08	1.8 × 10^−4^
26	17.59	296.2344	80286-58-4	Artemisic Acid	M − H_2_O − H, M − H, M + Cl	C_18_H_32_O_3_	−2.37	6.9 × 10^−4^
27	1.08	543.1320	439336	Galactomannan	M + K	C_18_H_32_O_16_	−0.30	4.6 × 10^−2^
28	1.39	158.1173	108866-42-8	2-Epilentiginosine	M + H	C_8_H_15_NO_2_	−1.69	1.6 × 10^−7^
29	12.41	446.1217	20633-67-4	Calycosin-7-*O*-Beta-d-Glucoside	M + H, M + Na	C_22_H_22_O_10_	0.97	1.6 × 10^−3^
30	12.61	477.1388	113235-89-5	7,2′-Dihydroxy-3′,4′-dimethoxyisoflavone 7-*O*-glucoside	M + H	C_23_H_24_O_11_	−0.78	5.3 × 10^−15^
31	13.47	563.1392	27661-51-4	Leucoside	M + H − H_2_O	C_26_H_28_O_15_	−0.57	8.2 × 10^−17^
32	14.07	430.1267	486-62-4	Ononin	M + H, M + Na	C_22_H_22_O_9_	0.73	4.3 × 10^−7^
33	14.12	287.0911	122587-87-5	(3R)-3′,8-Dihydroxyvestitol	M + H − H_2_O	C_16_H_16_O_6_	−0.98	6.9 × 10^−13^
34	14.27	316.0946	158991-20-9	Astragaluquinone	M + H, M + Na	C_17_H_16_O_6_	−0.26	3.9 × 10^−8^
35	14.41	462.1519	94367-42-7	Methylnissolin-3-*O*-glucoside	M + H, M + NH_4_, M + Na	C_23_H_26_O_10_	−1.59	9.2 × 10^−7^
36	14.44	300.0988	72026-91-6	Astraciceran	M + H − H_2_O, M +	C_17_H_16_O_5_	−3.15	1.7 × 10^−11^
37	15.00	314.0791	3301-49-3	Kumatakenin	M + H, M + Na, M + K	C_17_H_14_O_6_	0.18	1.9 × 10^−12^
38	15.22	472.3549	465-99-6	Hederagenin	M + H − H_2_O, M + H	C_30_H_48_O_4_	−0.67	2.2 × 10^−11^
39	15.24	302.1150	158784-72-6	8-Methoxyvestitol	M + H, M + Na	C_17_H_18_O_5_	−1.51	1.3 × 10^−6^
40	15.49	316.0946	69359-09-7	Pendulone	M + H−H_2_O, M H, M + Na	C_17_H_16_O_6_	−0.24	8.9 × 10^−4^
41	15.86	490.3650	78574-94-4	Cycloastragenol	M + H − H_2_O, M + Na	C_30_H_50_O_5_	−1.68	4.5 × 10^−10^
42	15.88	807.4503	84687-42-3	Astragaloside III	M + Na	C_41_H_68_O_14_	0.18	1.9 × 10^−9^
43	16.20	298.0842	740-33-0	Mosloflavone	M + H, M + Na	C_17_H_14_O_5_	0.34	4.2 × 10^−14^
44	16.22	274.2743	57-10-3	hexadecanoic acid	M + NH_4_	C_16_H_32_O_2_	1.05	2.4 × 10^−2^
45	16.22	301.1067	73340-41-7	3-Hydroxy-9,10-dimethoxyptercarpan	M + H	C_17_H_16_O_5_	−1.09	1.2 × 10^−6^
46	16.41	645.3974	101683491	Trigonoside I	M + Na	C_35_H_58_O_9_	0.12	2.2 × 10^−7^
47	16.44	620.3922	13943265	Cycloorbigenin 3-*O*-beta-d-xylopyranoside	M + H − H_2_O, M + Na	C_35_H_56_O_9_	−0.37	8.5 × 10^−7^
48	17.03	473.3617	86541-79-9	Astragenol	M + H − H_2_O	C_30_H_50_O_5_	−1.66	4.0 × 10^−5^
49	17.07	687.4079	133550435	Cyclogaleginoside A	M + Na	C_37_H_60_O_10_	0.11	4.0 × 10^−8^
50	18.42	279.2315	463-40-1	Linolenic acid	M + H	C_18_H_30_O_2_	−1.36	7.0 × 10^−5^
51	19.93	263.2367	693-77-6	Octadeca-9,12-dienoic acid	M + H − H_2_O	C_18_H_32_O_2_	−0.73	5.9 × 10^−3^
52	21.68	409.3823	545-47-1	Lupeol	M + H − H_2_O	C_30_H_50_O	−1.45	2.1 × 10^−2^
53	21.93	397.3826	83-46-5	(−)-beta-Sitosterol	M + H − H_2_O	C_29_H_50_O	−0.76	1.8 × 10^−2^

## Supplementary Materials

The following are available online. Figure S1: The experimental procedures for chemical discrimination of MG and MJ. Figure S2: The base peak chromatograms in positive mode and negative mode. Figure S3: The VIP and PCA plots between MG and MJ. Figure S4: The detail MS and MS/MS information of Calycosin. Figure S5: The heat map analysis and detail relative content comparison of chemical markers between MG and MJ. Peak 1-53: The MS/MS maps of discriminating compounds between MG and MJ.
