# Supplementary figures and images for: Chemical Discrimination of Astragalus mongholicus and Astragalus membranaceus Based on Metabolomics Using UHPLC-ESI-Q-TOF-MS/MS Approach
Source: Molecules. 2019 Nov 9;24(22):4064. doi: 10.3390/molecules24224064 (PMC6891664; doi:10.3390/molecules24224064)

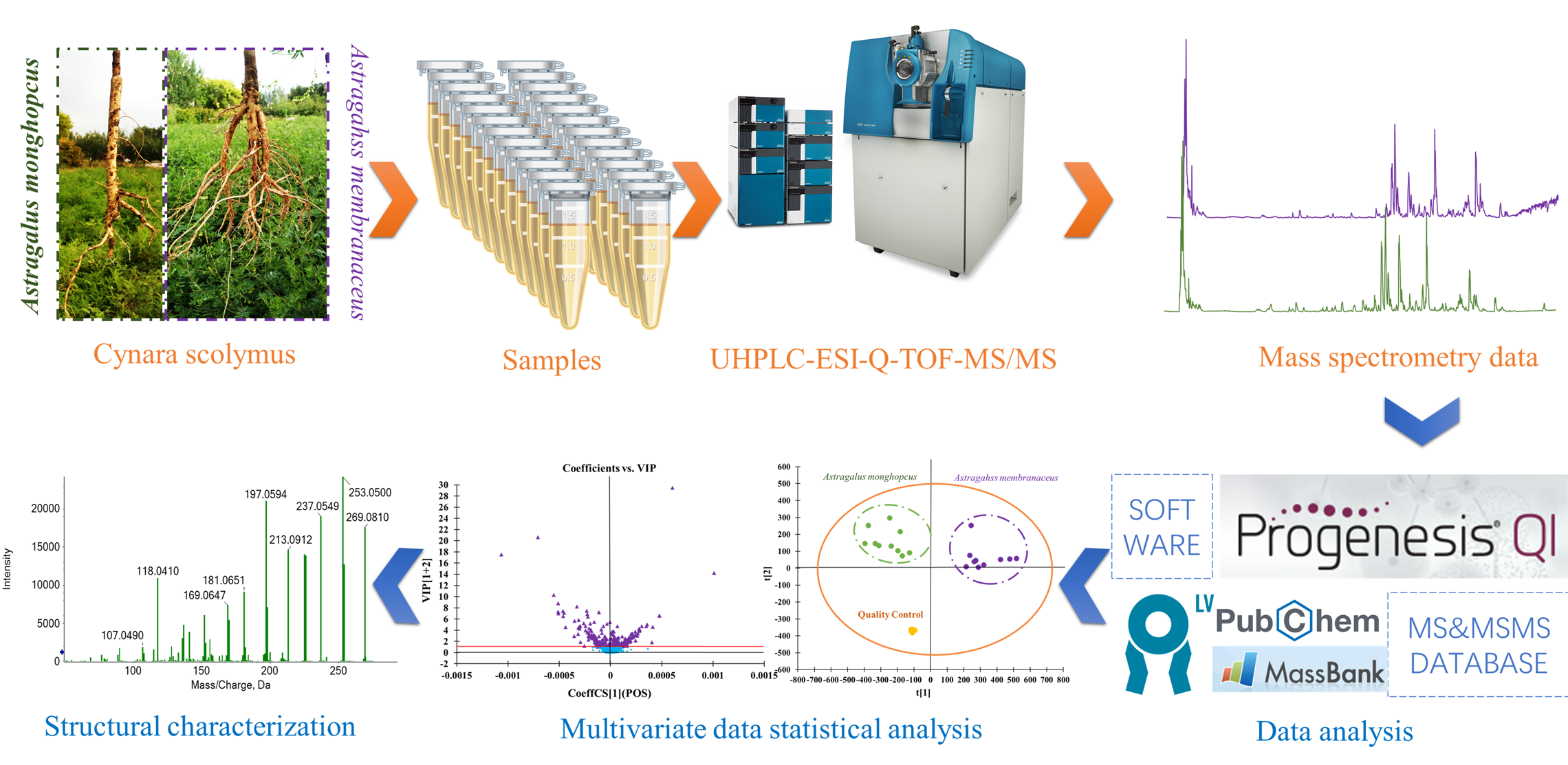

Supplement: Supplementary file 1 [file molecules-24-04064-s001.zip › Supporting information/Figure S1.tif]

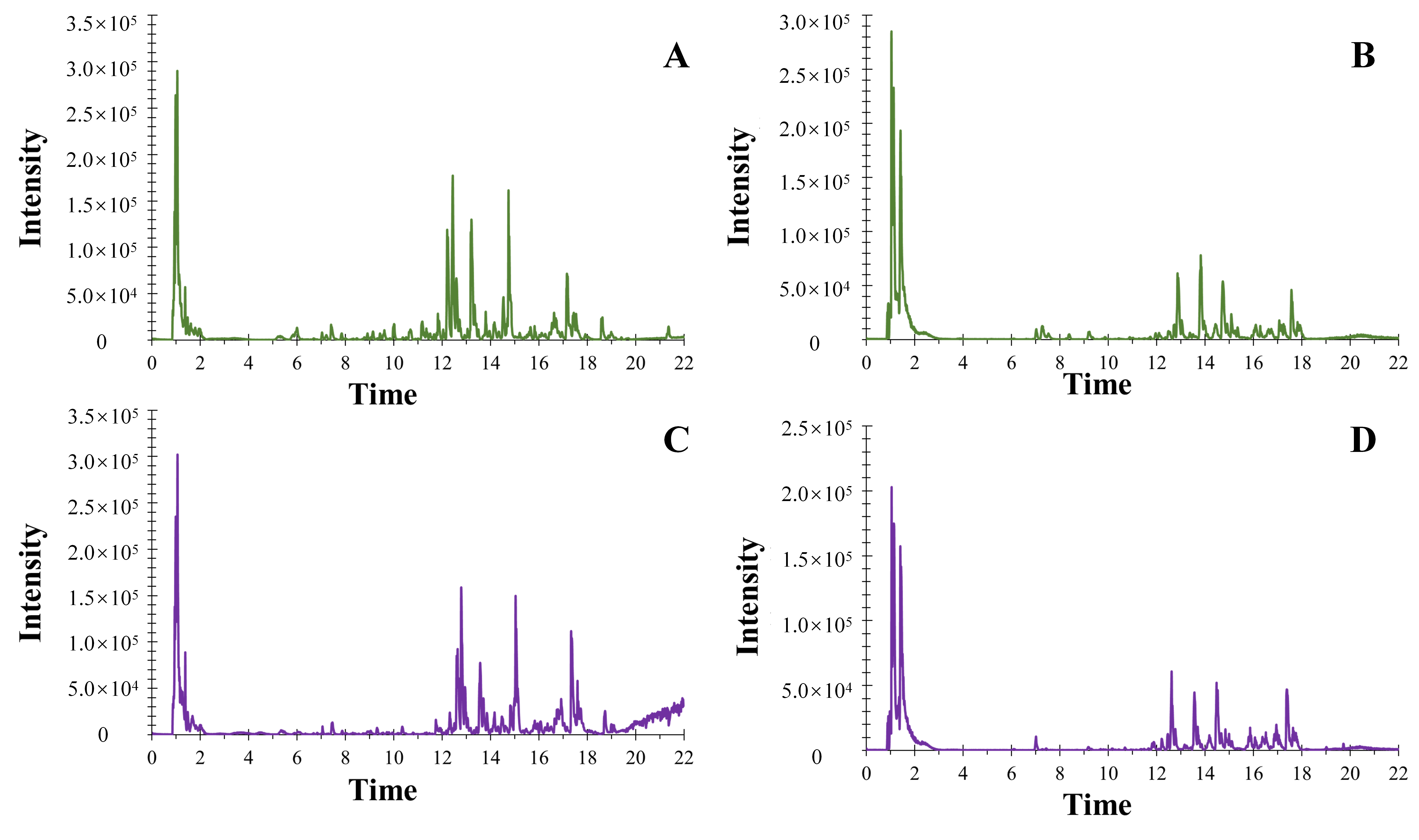

Supplement: Supplementary file 1 [file molecules-24-04064-s001.zip › Supporting information/Figure S2.tif]

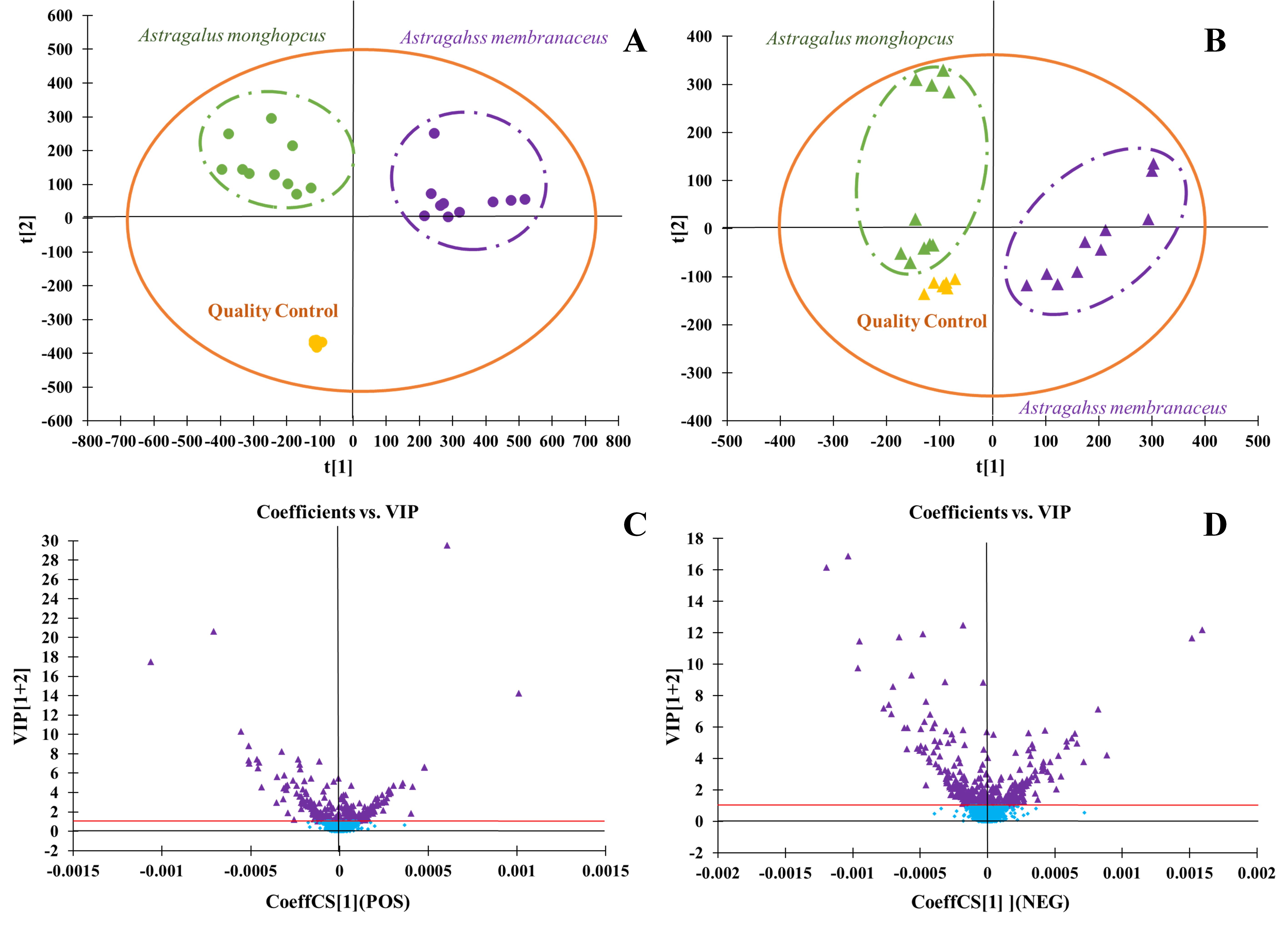

Supplement: Supplementary file 1 [file molecules-24-04064-s001.zip › Supporting information/Figure S3.tif]

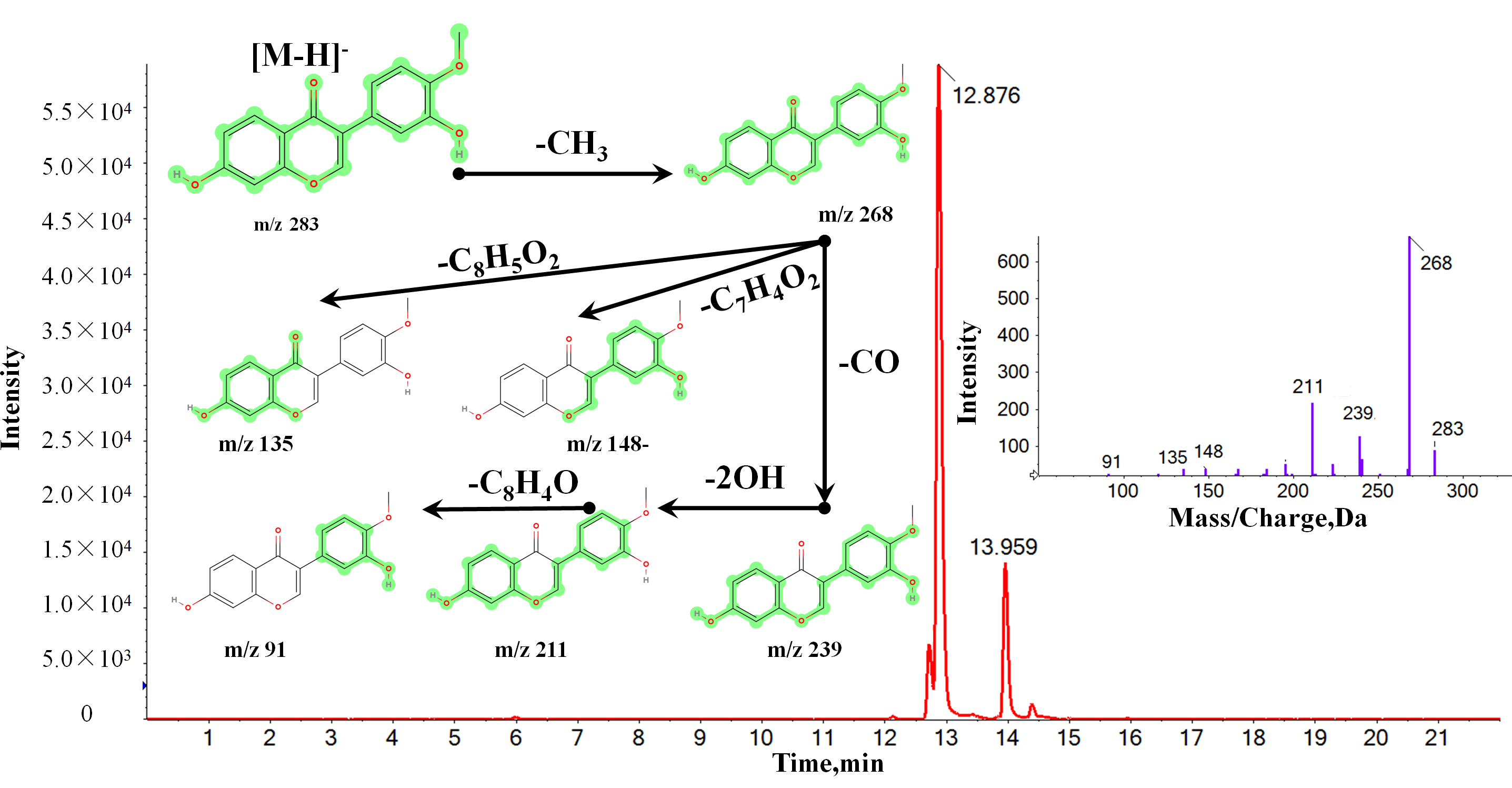

Supplement: Supplementary file 1 [file molecules-24-04064-s001.zip › Supporting information/Figure S4.tif]

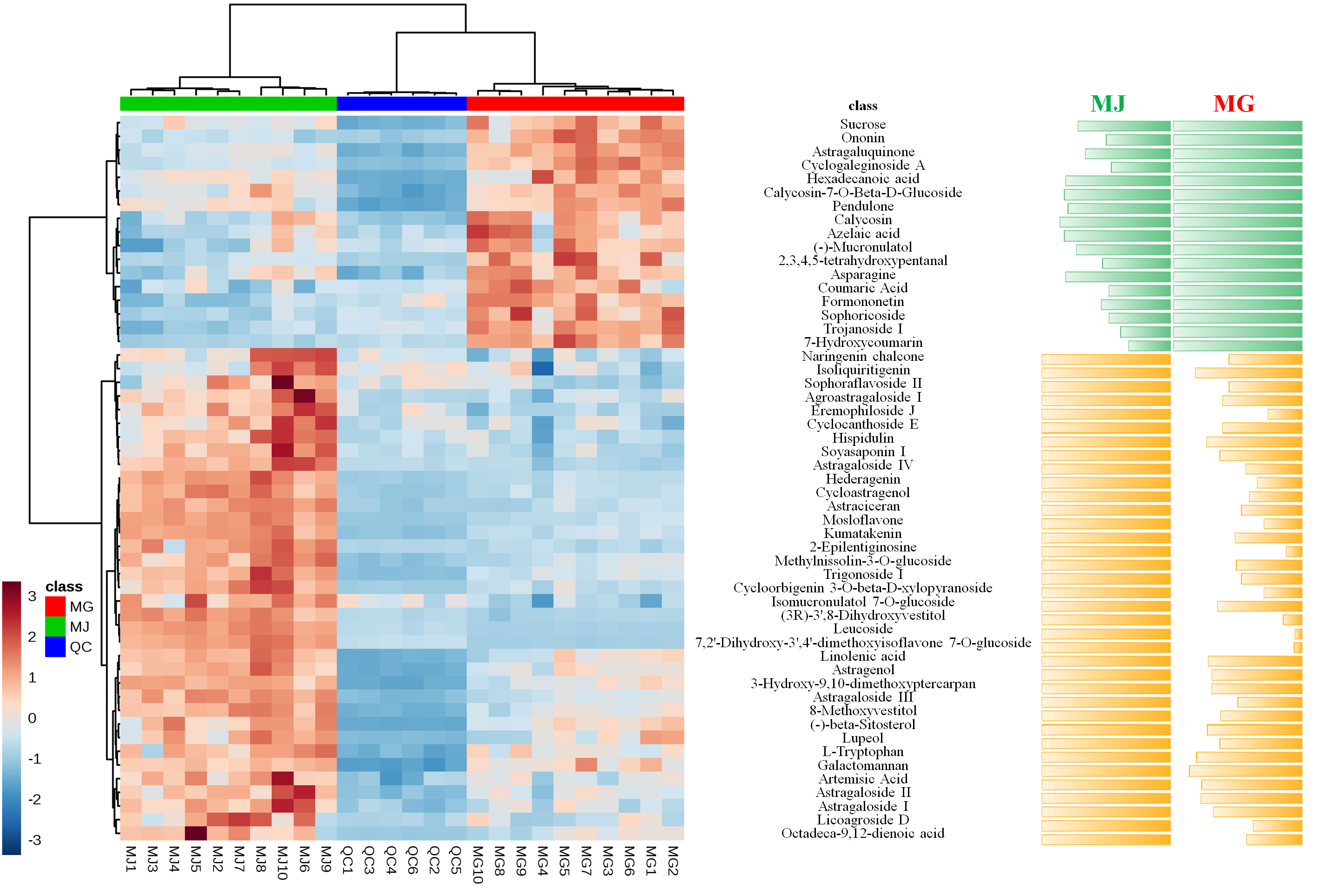

Supplement: Supplementary file 1 [file molecules-24-04064-s001.zip › Supporting information/Figure S5.tif]

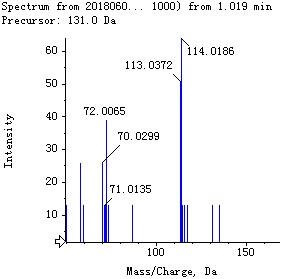

Supplement: Supplementary file 1 [file molecules-24-04064-s001.zip › Supporting information/Peak 1-53/1.jpg]

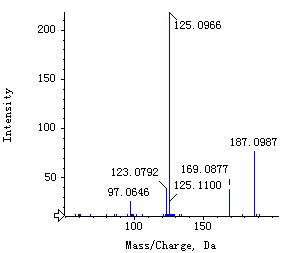

Supplement: Supplementary file 1 [file molecules-24-04064-s001.zip › Supporting information/Peak 1-53/10.jpg]

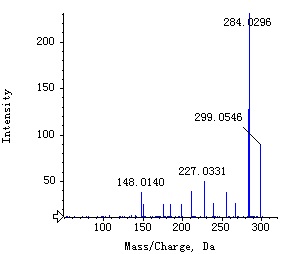

Supplement: Supplementary file 1 [file molecules-24-04064-s001.zip › Supporting information/Peak 1-53/11.jpg]

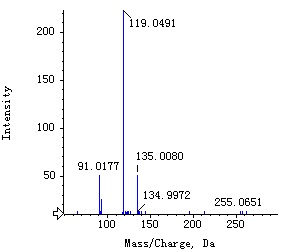

Supplement: Supplementary file 1 [file molecules-24-04064-s001.zip › Supporting information/Peak 1-53/12.jpg]

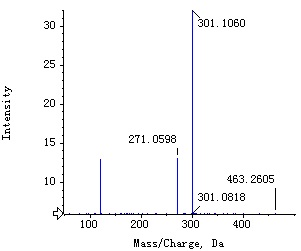

Supplement: Supplementary file 1 [file molecules-24-04064-s001.zip › Supporting information/Peak 1-53/13.jpg]

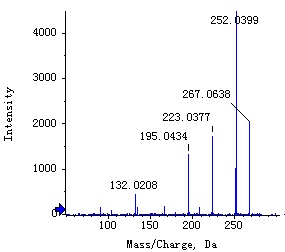

Supplement: Supplementary file 1 [file molecules-24-04064-s001.zip › Supporting information/Peak 1-53/14.jpg]

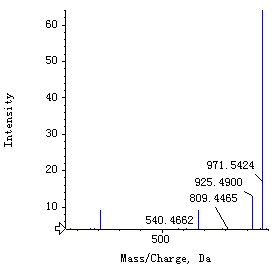

Supplement: Supplementary file 1 [file molecules-24-04064-s001.zip › Supporting information/Peak 1-53/15.jpg]

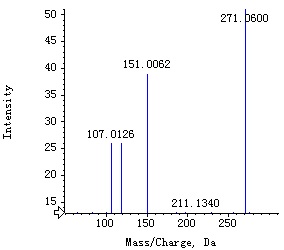

Supplement: Supplementary file 1 [file molecules-24-04064-s001.zip › Supporting information/Peak 1-53/16.jpg]

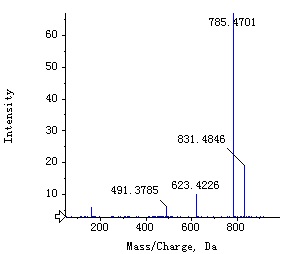

Supplement: Supplementary file 1 [file molecules-24-04064-s001.zip › Supporting information/Peak 1-53/17.jpg]

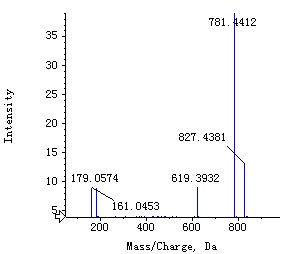

Supplement: Supplementary file 1 [file molecules-24-04064-s001.zip › Supporting information/Peak 1-53/18.jpg]

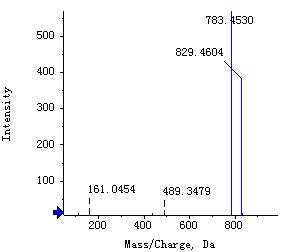

Supplement: Supplementary file 1 [file molecules-24-04064-s001.zip › Supporting information/Peak 1-53/19.jpg]

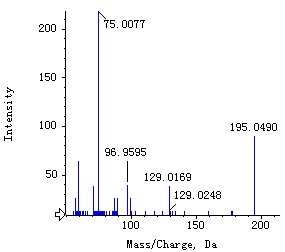

Supplement: Supplementary file 1 [file molecules-24-04064-s001.zip › Supporting information/Peak 1-53/2.jpg]

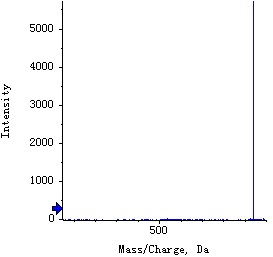

Supplement: Supplementary file 1 [file molecules-24-04064-s001.zip › Supporting information/Peak 1-53/20.jpg]

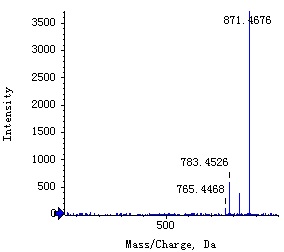

Supplement: Supplementary file 1 [file molecules-24-04064-s001.zip › Supporting information/Peak 1-53/21.jpg]

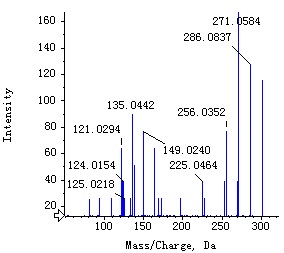

Supplement: Supplementary file 1 [file molecules-24-04064-s001.zip › Supporting information/Peak 1-53/22.jpg]

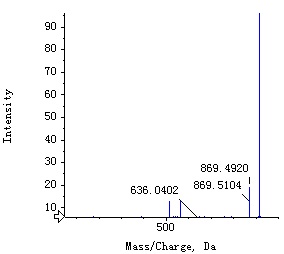

Supplement: Supplementary file 1 [file molecules-24-04064-s001.zip › Supporting information/Peak 1-53/23.jpg]

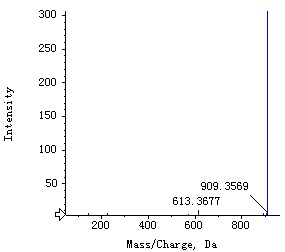

Supplement: Supplementary file 1 [file molecules-24-04064-s001.zip › Supporting information/Peak 1-53/24.jpg]

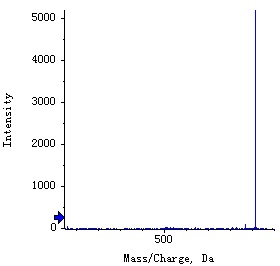

Supplement: Supplementary file 1 [file molecules-24-04064-s001.zip › Supporting information/Peak 1-53/25.jpg]

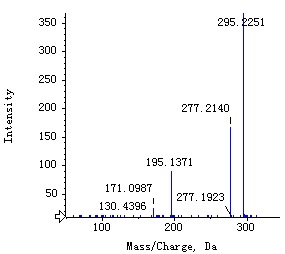

Supplement: Supplementary file 1 [file molecules-24-04064-s001.zip › Supporting information/Peak 1-53/26.jpg]

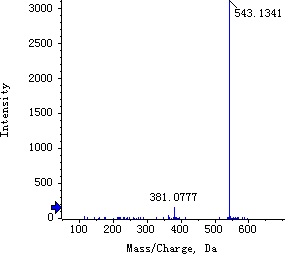

Supplement: Supplementary file 1 [file molecules-24-04064-s001.zip › Supporting information/Peak 1-53/27.jpg]

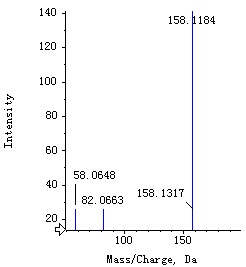

Supplement: Supplementary file 1 [file molecules-24-04064-s001.zip › Supporting information/Peak 1-53/28.jpg]

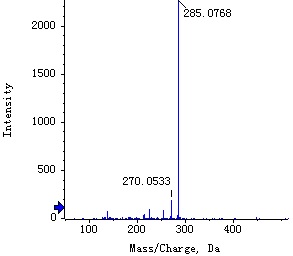

Supplement: Supplementary file 1 [file molecules-24-04064-s001.zip › Supporting information/Peak 1-53/29.jpg]

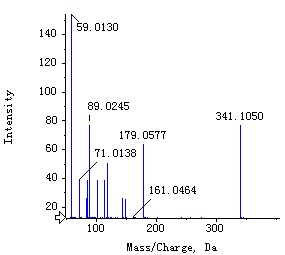

Supplement: Supplementary file 1 [file molecules-24-04064-s001.zip › Supporting information/Peak 1-53/3.jpg]

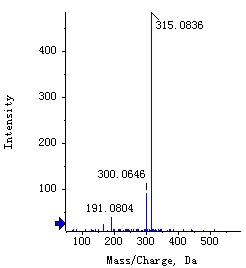

Supplement: Supplementary file 1 [file molecules-24-04064-s001.zip › Supporting information/Peak 1-53/30.jpg]

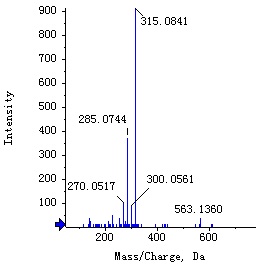

Supplement: Supplementary file 1 [file molecules-24-04064-s001.zip › Supporting information/Peak 1-53/31.jpg]

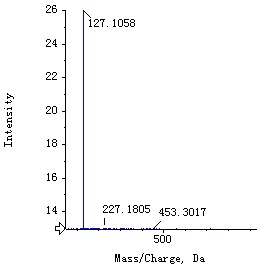

Supplement: Supplementary file 1 [file molecules-24-04064-s001.zip › Supporting information/Peak 1-53/32.jpg]

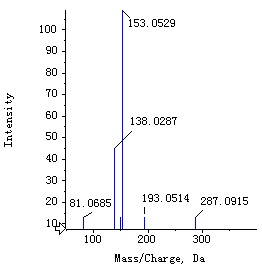

Supplement: Supplementary file 1 [file molecules-24-04064-s001.zip › Supporting information/Peak 1-53/33.jpg]

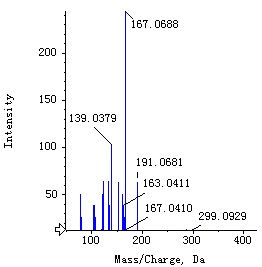

Supplement: Supplementary file 1 [file molecules-24-04064-s001.zip › Supporting information/Peak 1-53/34.jpg]

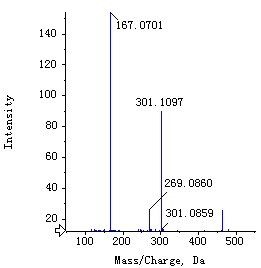

Supplement: Supplementary file 1 [file molecules-24-04064-s001.zip › Supporting information/Peak 1-53/35.jpg]

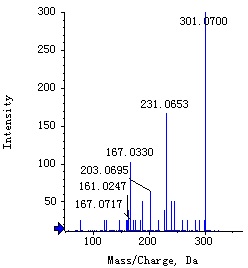

Supplement: Supplementary file 1 [file molecules-24-04064-s001.zip › Supporting information/Peak 1-53/36.jpg]

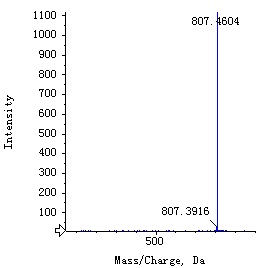

Supplement: Supplementary file 1 [file molecules-24-04064-s001.zip › Supporting information/Peak 1-53/37.jpg]

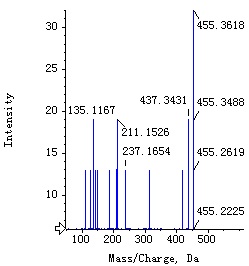

Supplement: Supplementary file 1 [file molecules-24-04064-s001.zip › Supporting information/Peak 1-53/38.jpg]

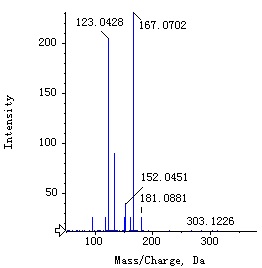

Supplement: Supplementary file 1 [file molecules-24-04064-s001.zip › Supporting information/Peak 1-53/39.jpg]

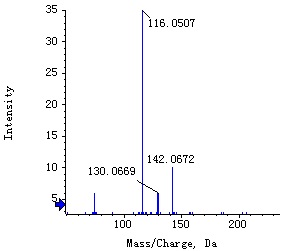

Supplement: Supplementary file 1 [file molecules-24-04064-s001.zip › Supporting information/Peak 1-53/4.jpg]

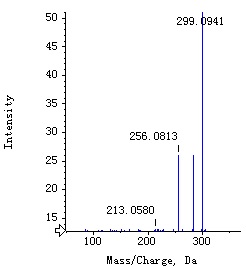

Supplement: Supplementary file 1 [file molecules-24-04064-s001.zip › Supporting information/Peak 1-53/40.jpg]

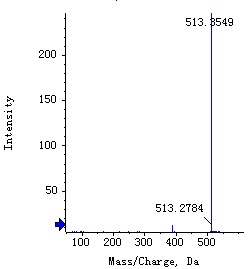

Supplement: Supplementary file 1 [file molecules-24-04064-s001.zip › Supporting information/Peak 1-53/41.jpg]

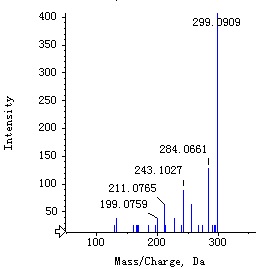

Supplement: Supplementary file 1 [file molecules-24-04064-s001.zip › Supporting information/Peak 1-53/43.jpg]

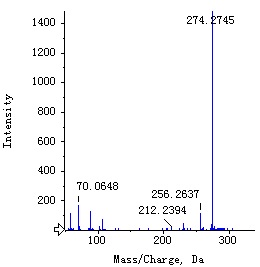

Supplement: Supplementary file 1 [file molecules-24-04064-s001.zip › Supporting information/Peak 1-53/44.jpg]

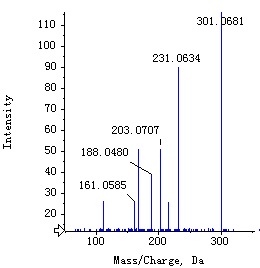

Supplement: Supplementary file 1 [file molecules-24-04064-s001.zip › Supporting information/Peak 1-53/45.jpg]

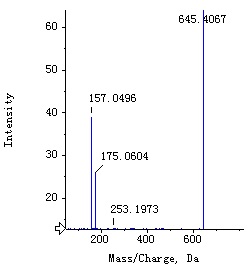

Supplement: Supplementary file 1 [file molecules-24-04064-s001.zip › Supporting information/Peak 1-53/46.jpg]

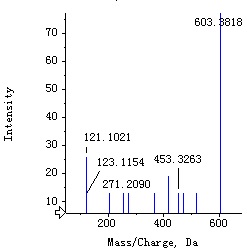

Supplement: Supplementary file 1 [file molecules-24-04064-s001.zip › Supporting information/Peak 1-53/47.jpg]

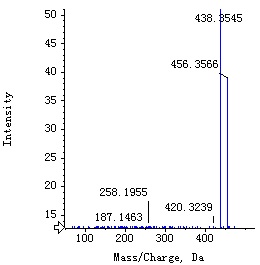

Supplement: Supplementary file 1 [file molecules-24-04064-s001.zip › Supporting information/Peak 1-53/48.jpg]

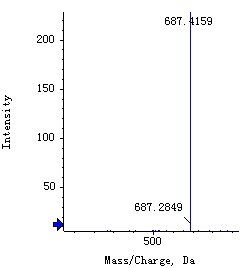

Supplement: Supplementary file 1 [file molecules-24-04064-s001.zip › Supporting information/Peak 1-53/49.jpg]

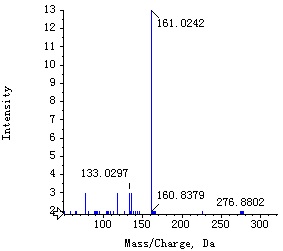

Supplement: Supplementary file 1 [file molecules-24-04064-s001.zip › Supporting information/Peak 1-53/5.jpg]

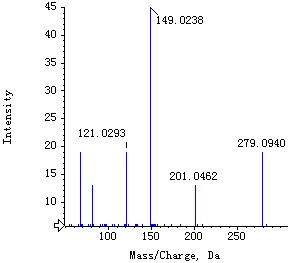

Supplement: Supplementary file 1 [file molecules-24-04064-s001.zip › Supporting information/Peak 1-53/50.jpg]

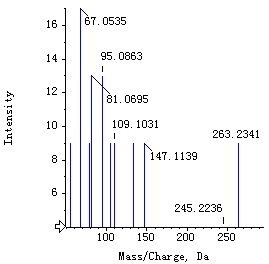

Supplement: Supplementary file 1 [file molecules-24-04064-s001.zip › Supporting information/Peak 1-53/51.jpg]

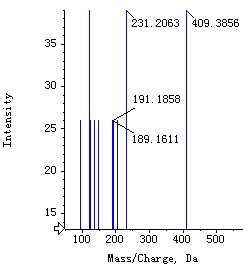

Supplement: Supplementary file 1 [file molecules-24-04064-s001.zip › Supporting information/Peak 1-53/52.jpg]

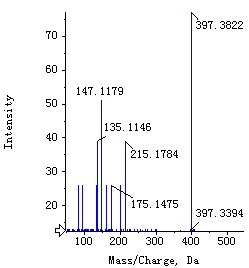

Supplement: Supplementary file 1 [file molecules-24-04064-s001.zip › Supporting information/Peak 1-53/53.jpg]

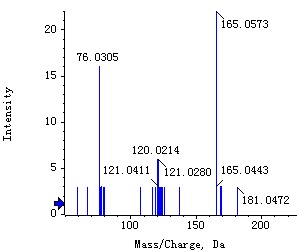

Supplement: Supplementary file 1 [file molecules-24-04064-s001.zip › Supporting information/Peak 1-53/6.jpg]

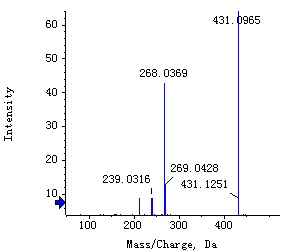

Supplement: Supplementary file 1 [file molecules-24-04064-s001.zip › Supporting information/Peak 1-53/7.jpg]

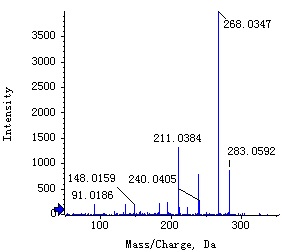

Supplement: Supplementary file 1 [file molecules-24-04064-s001.zip › Supporting information/Peak 1-53/8.jpg]

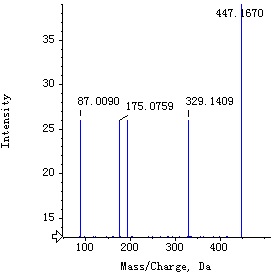

Supplement: Supplementary file 1 [file molecules-24-04064-s001.zip › Supporting information/Peak 1-53/9.jpg]
